# Investigating the mechanical properties and crashworthiness of hybrid PLA/GFRP composites fabricated using FDM-filament winding

**DOI:** 10.1016/j.heliyon.2024.e39062

**Published:** 2024-10-09

**Authors:** Ariyana Dwiputra Nugraha, Rahmad Kuncoro Adi, Vishnu Vijay Kumar, Arif Kusumawanto, Budi Prawara, Endro Junianto, Muhammad Fathul Hikmawan, Muhammad Akhsin Muflikhun

**Affiliations:** a PLN Research Institute, Jakarta, 12760, Indonesia; b Department of Mechanical and Industrial Engineering, Universitas Gadjah Mada, 55281, Indonesia; c Universitas Muhammadiyah Yogyakarta, 55183, Indonesia; d Structural Engineering, Division of Engineering, New York University Abu Dhabi (NYUAD), PO Box 129188, Abu Dhabi, United Arab Emirates; e Department of Architecture and Planning, Universitas Gadjah Mada, Jalan Grafika No. 2, Yogyakarta, 55281, Indonesia; f Research Center for Electrical Power and Mechatronics—National Research and Innovation Agency (BRIN), Jl. Sangkuriang, Dago, Kecamatan Coblong, Kota Bandung, Jawa Barat, 40135, Indonesia; g Center for Energy Studies (PSE), Universitas Gadjah Mada, Indonesia

**Keywords:** Crashworthiness, Composite, Filament winding, Additive manufacturing

## Abstract

The failure and crashworthiness performance of hybrid Polylactide (PLA)/Glass Fiber Reinforced Polymer (GFRP) composite tubes subjected to axial and radial quasistatic compression loads were investigated in the present study. The composite tubes are fabricated using hand lay-up and Fused Deposition Modeling (FDM)-filament winding processes. The crashworthiness performance was studied using parameters such as the peak load, energy absorption, crush load, crushing load efficiency, and specific energy absorption. The highest peak loads from the compression test results in the axial and radial directions are calculated using variations in the filament winding PLA/GFRP (FPG) specimen, whose values were 16130.50 N and 12077.33 N, respectively. The highest mean crush load is found in the material variation with the FPG specimen, which is 5734.43 J/mm for axial loading and 4886.75 J/mm for radial loading. The highest energy absorption (EA) value is found in the material variation with the FPG specimen, which is 262.18 J for axial direction loading and 94.02 J for radial direction loading. The highest specific energy absorption (SEA) value is found in the material variation with the specimen Filament Winding PLA/GFRP, which is 16.92 J/g for axial loading and 10.98 J/g for radial loading. There are three main types of failure of the composite: matrix cracking, fiber damage, and folding. The study showed that by combining Additive Manufacturing and composite lamination, the hybrid PLA/GFRP specimen outperformed the existing structures.

## 1 Introduction

The crashworthiness of structures is a critical factor in providing better protection to individuals during accidents [1,2]. Recently, there has been a growing demand for more crash-resistant materials in the automotive and aviation industries [[3], [4], [5]]. The necessity for thin-walled structures, such as aircraft fuselages and steering columns, to demonstrate superior energy absorption capabilities during impact events has driven the demand for materials with enhanced crashworthiness performance [[6], [7], [8]]. Researchers have conducted experiments to enhance the crashworthiness of these thin-walled structures by altering their geometrical configurations and designs [9,10]. Moreover, the aviation and automotive industries have shown growing interest in employing lightweight, energy-absorbing materials, as these sectors face stringent requirements to meet higher eligibility standards [[11], [12], [13]]. Consequently, reducing the weight of a structure without compromising its crashworthiness performance has become essential [14]. With the increasing adoption of additive manufacturing techniques, FDM-filament winding has been employed to fabricate complex designs with excellent properties. Compared to conventional methods, the filament winding process offers high production rates, consistent product quality, and repeatable manufacturing processes [15]. The FDM-filament winding process used in this study can be considered a hybrid manufacturing technique. This method combines additive manufacturing (FDM) with traditional filament winding, enabling the production of composite structures that integrate both PLA and GFRP materials. This combination allows for the creation of more complex structures with enhanced mechanical properties, leveraging the benefits of both FDM and filament winding processes [16,17].

In this context, polylactic acid (PLA) has emerged as a suitable material option due to the advent of additive manufacturing technologies. PLA is a plastic material with relatively low production costs and natural degradability [18]. Improving the mechanical properties of PLA materials has become a widely discussed issue in academia and industry [19,20]. Several steps have been implemented, including the use of fillers [21] and nanomaterials [22,23], the blending of PLA material with other materials [24], and the use of fibers [25]. Introducing fibers is a practical approach to improving the mechanical properties of PLA [26]. Generally, natural fibers are more environmentally friendly [[27], [28], [29]] than synthetic fibers, but their strength is still relatively low [30]. Consequently, to enhance the mechanical properties of PLA materials, reinforcing synthetic fibers with higher strength are incorporated. The most widely employed synthetic fiber for this purpose is glass fibers [31]. They act as good reinforcements, have good mechanical properties and high rigidity, and are relatively inexpensive [[32], [33], [34]].

The choice of PLA/GFRP as the material for this study is driven by their complementary properties. PLA is widely recognized for its low cost, ease of production, and biodegradability, making it an attractive material for environmentally sustainable applications. However, its mechanical strength is relatively low. On the other hand, GFRP (Glass Fiber Reinforced Polymer) offers high mechanical strength and rigidity, which can significantly enhance the performance of PLA when used in hybrid structures [35,36]. These hybrid composites are particularly suitable for applications in the automotive and aerospace industries, where lightweight materials with high crashworthiness and energy absorption capabilities are in demand [14,24].

The damage mechanism of metallic materials is less complex than that of composite materials. Composite damage occurs due to fiber breaking, buckling, matrix cracking, delamination, and fiber debonding [15,[37], [38], [39], [40]]. The energy absorption characteristics of composites are determined by the type of material, geometric structure, loading conditions, and winding angle [41]. Previous researchers have used several materials to make hybrid composites, including PLA [42,43], ABS [44,45], and nylon [46]. Research conducted by Usun et al. with PLA as the matrix and carbon fiber as the reinforcement showed that a hybrid composite with 40 % CF had higher tensile and bending strengths than a hybrid composite with 22 % CF and 33 % CF [47]. Another study conducted by Muller et al. with PLA as the matrix and pinewood and cork as reinforcements showed that the tensile strength of PLA plus cork and pinewood increased by 31 % and 19.5 %, respectively, compared to that of pure PLA [48]. Prajapati et al. used nylon as a matrix and glass fiber as a reinforcement to make hybrid composites using an FDM machine. The results obtained from this study show that the impact strength of the composite increased as the number of layers of reinforcement increased. The highest impact value was found for layer 119 fibers, with an impact value of 2448.34 J/m, while the lowest impact value was found for layer 29 fibers, with an impact value of 1566.03 J/m [49]. Continuous fiber reinforcement with PLA can be employed as an effective method for improving crashworthiness and energy absorption [50].

Earlier studies employed quasi-static compression testing to evaluate the crashworthiness characteristics of materials. Bakar et al. [51] conducted quasi-static compression tests to analyze the energy absorption behavior of hybrid kenaf/glass fiber-reinforced epoxy composites with three different stacking sequences. Compared with the glass fiber-reinforced epoxy tube, the hybrid specimen with the highest fiber volume fraction significantly increased the initial peak load by 28 % and the amount of energy absorbed by 68 %. Hu et al. [52] also performed axial quasistatic compression tests to investigate the energy absorption characteristics of foam-filled tritubes. The results of an experiment they conducted showed that a foam-filled tritube has better energy absorption performance than other tubes. Ozbek et al. [53] experimented to explore the failure modes and crashworthiness characteristics of intraply hybridized fiber-reinforced pipes (FRPs) made of basalt and glass fiber reinforcements subjected to quasistatic compression loading. From the aspect of fiber orientation, a decrease in the winding angle resulted in a significant increase in the energy absorption capability of all pipes. The pipes made from glass fibers showed the best value of the specific energy absorption characteristic. Several studies have examined the effectiveness of crashworthiness employing quasistatic compression [[54], [55], [56]]. However, there is still a lack of comprehensive research on hybrid PLA/GFRP specimens in the open literature, especially experimental exploration.

This study is structured as follows: Section 2 discusses the materials and methods used in fabricating the hybrid PLA/GFRP composites, including the FDM-filament winding and hand lay-up techniques. Section 3 presents the experimental results, focusing on the mechanical properties, crashworthiness performance, and failure analysis of the specimens. Section 4 provides a detailed discussion of the results, comparing the performance of different material configurations. Finally, Section 5 concludes the study by summarizing the key findings and suggesting potential future research directions. The current study investigated the failure and crashworthiness performance of a PLA/GFRP hybrid composite material under quasistatic compression testing. The specimens were fabricated using the additive manufacturing technique of FDM-filament winding. The performance was evaluated using quasistatic compression tests with axial and radial loading, and hardness, density, and surface roughness evaluations were also carried out. This study provides insight into the performance and failure of hybrid additive-fabricated PLA/GFRP composites.

## 2 Materials and methods

### 2.1 Materials

The materials used for fabricating the specimens included e-SUN PLA, properties given in Table 1 and fiberglass roving (ER 2400 TEX). Epoxy resin bisphenol A (viscosity 13000 MPas, density 1.17 g/cm^3^) and the hardener EPH 555 (density 1.01 g/cm^3^) were obtained from PT. Justus Kimia Raya [57,58].

**Table 1:** Properties of the PLA filament.

Properties	Value	Unit
Print Temp	205–225	^0^C
Bed Temp	60–80	^0^C
Density	1.25	g/cm^3^
Melt Flow Index	4	g/10 min
Tensile Strength	65	MPa
Elongation at Break	12	%
Flexural Strength	75	MPa
Flexural Modulus	2102	MPa
Impact Strength	8.5	KJ/m^2^

### 2.2 PLA tube preparation

The PLA tubes were designed using Autodesk Inventor 2021 software. The dimensions of the tube are based on the size of the mandrel found on the filament winding machine.

**Fig. 1:**
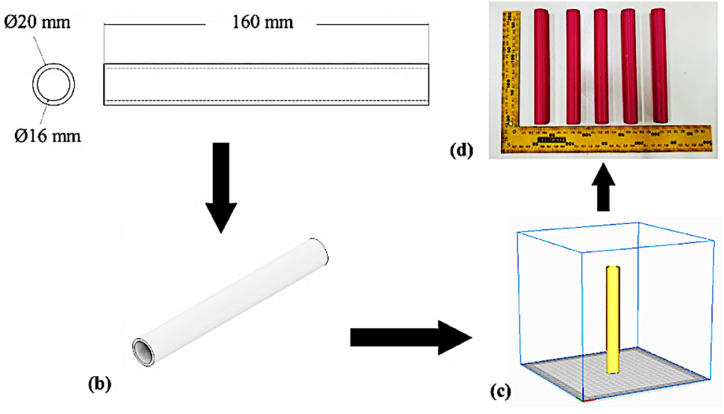
Tube manufacturing process: (a) 2D model, (b) 3D model, (c) slicing process, (d) finished product.

The 3D printing machine used for fabrication was an Anycubic i3 Mega S/Pro. The specifications of the 3D printer used and the detailed setup of the 3D printer machine in the present study are shown in Table 2, Table 3. A schematic representation of the tube manufacturing process is shown in Fig. 1(a–d). Fig. 1(a) presents the 2D model of the tube dimensions and geometry. Fig. 1(b) shows the 3D model for the printing process. Fig. 1(c) illustrates the slicing process where the 3D model is divided into layers for printing. Fig. 1(d) displays the final product after the printing process is completed.

**Table 2:** Specification 3D printer Anycubic i3 Mega S/Pro.

Specification	Value	Unit
Printing Technology	Fused Deposition Modeling (FDM)	–
Build Volume	210 × 210 x 205	mm
Min. Layer Height	100	micron
Extruder Type	Single	–
Nozzle Size	0.4	Mm
Max. Extruder Temp	275	^0^C
Max. Heated Bed Temp	100	^0^C
Supported Print Materials	ABS, Composite, HIPS, PLA, PETG, TPU	–

**Table 3:** Set up 3D printing.

Setting Properties	Value	Unit
Layer height	0.1	mm
Wall thickness	0.8	mm
Infill pattern	Concentric	–
Infill density	100	%
Printing temperature	200	°C
Build plate temperature	60	°C
Print speed	50	mm/s
Retraction	On	–
Build plate adhesion type	Skirt	–

### 2.3 Composite fabrication

There are three different types of composite tubes: plain PLA, plain GFRP only, and a combination of PLA and GFRP composite (hybrid). FDM-filament winding and hand lay-up methods were used to fabricate the GFRP and the hybrid PLA/GFRP composite. During the tube fabrication process, GFRP material and a combination of PLA and GFRP are installed on the mandrel in the filament winding machine. After the composite tube manufacturing process, the tube was cut to a length of 45.72 mm. The specimen codes are listed in Table 4.

**Table 4:** Specimen codes.

No	Methods	Material	Code
**1**	Additive Manufacturing	PLA	PLA

**2**	Hand Lay-Up	GFRP	HG
**3**		PLA/GFRP	HPG
**4**	Filament Winding	GFRP	FG
**5**		PLA/GFRP	FPG

#### 2.3.1 Filament winding

**Fig. 2:**
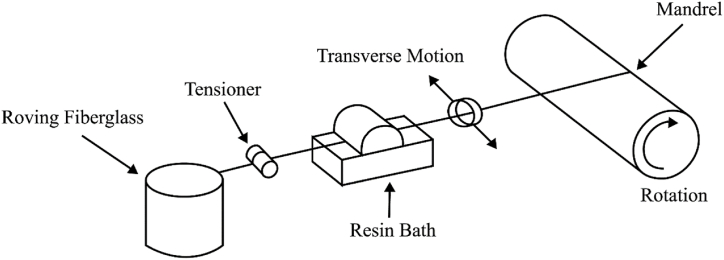
Schematic of the filament winding manufacturing process.

The fabrication of composite tubes in this study was carried out using filament winding and lay-up methods. A schematic of the process of making composite tubes using the filament winding method is shown in Fig. 2 [59]. The first step in the process is to coat the mandrel with antistatic plastic. The mandrel of the filament winding machine is made of steel rods. The following process involves the addition of a 4:1 mixture of winding glass fibers to the resin-hardener mixture. During the fabrication of the hybrid PLA/GFRP material arrangement. The PLA tube is attached to a rotating mandrel, which is then subjected to the glass fiber winding process.

#### 2.3.2 Hand lay-up

Another method of fabricating composite tubes in this study is the hand lay-up technique. Composite tubes are made using the lay-up method by coating the mandrel with static plastic. The next process in the manufacturing of composite tubes with a GFRP material arrangement is to laminate glass fibers using a brush.

**Fig. 3:**
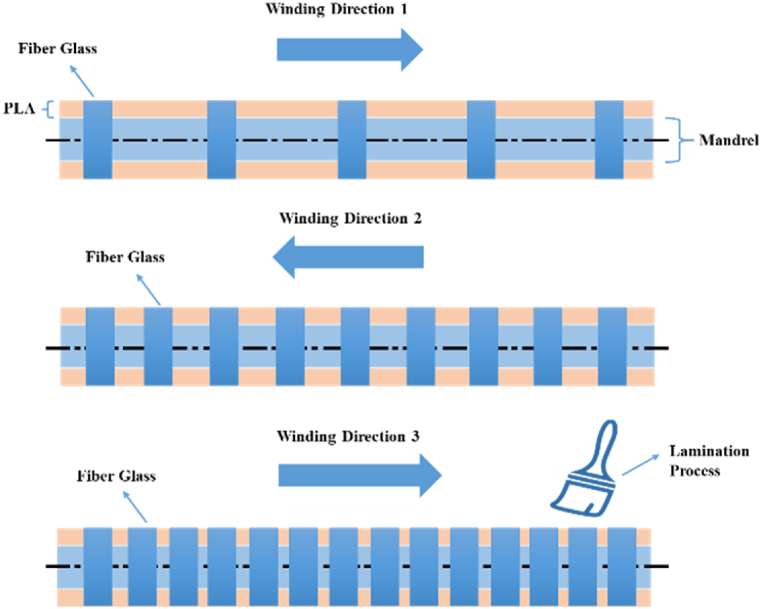
Lay-up method for the lamination process.

In the combined arrangement of the PLA/GFRP hybrid composite, the PLA material is mounted on a rotating mandrel, which is then subjected to the glass fiber winding process and the lamination process using a brush. The mixture of resin and hardener used was 4:1. The winding is repeated three times, as shown in Fig. 3.

### 2.4 Mechanical testing

**Fig. 4:**
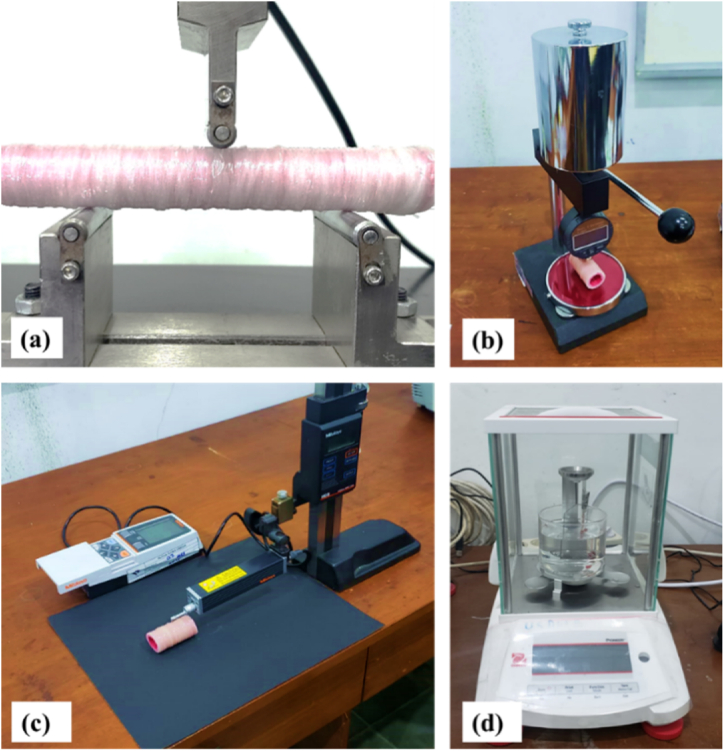
**(a)** Bending test, **(b)** Shore D hardness test, **(c)** surface roughness test, and **(d)** density test.

The properties of the PLA, GFRP, and PLA/GFRP hybrids were measured via compression tests, three-point quasistatic tests, hardness tests, surface roughness tests, and density tests, as shown in Fig. 4(a–d).

#### 2.4.1 Density test

Density testing was carried out using the OHAUS machine for each specimen tested. The specimens tested included PLA, GFRP, and hybrid PLA/GFRP. The size of the specimen used was determined according to the ASTM D 792 standard [60]. The tested material was cut into small pieces measuring 1–5 g.

#### 2.4.2 Compression test

**Fig. 5:**
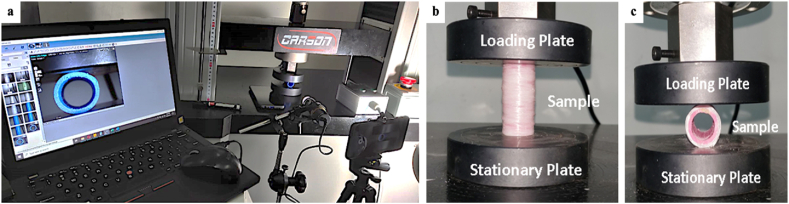
Schematic of the compression test process: **(a)** experimental setup, **(b)** axial testing, and **(c)** radial testing.

The compression test was carried out using a Carson 50 kN Universal Testing Machine (UTM) axially and radially according to the ASTM D 2412-02 [61] standards. A representation of the compression test process is shown in Fig. 5(a–c). The samples were placed on the fixed lower platen, and the moving upper platen was set at a constant crosshead speed of 2 mm/min. The load and displacement of the loading plate during the pressing process are recorded automatically by the data acquisition system. To evaluate the damage due to crushing of the specimen structure, three iterations of the test were carried out to ensure the experimental reliability and repeatability of the results.

To evaluate the crashworthiness performance of the composite tube under quasistatic compression loading, the following parameters are evaluated:

The peak load usually indicates the highest load point in the postcrushing zone. The mean load, known as P_mean_, is defined as the total absorbed energy (AE), with a force value (P) at each compression distance. Therefore, P_mean_ can be calculated using Eq. (1).

(1) $$P_{\text{mean}}=\text{AE}/L$$

The energy absorbed (EA) is the force at each compression distance, which is evaluated as the area under the load‒displacement curve. EA can be calculated using Eq. (2).

(2) $$\text{EA}=\int_{0}^{\Delta L}P\left(L\right)\;\text{dL}$$

The crashworthiness properties of a composite tube structure can be described as specific energy absorption (SEA). d is calculated as the energy absorbed per unit of crushed specimen, where M is the crushed specimen mass. SEA can be calculated using Eq. (3).

(3) $$\text{SEA}=\frac{\int_{0}^{\Delta L}P\left(L\right)\;\text{dL}}{M}$$

The crushing load efficiency (CLE), which is the percentage ratio of Pmean to Pmax, is a stable crashworthiness characteristic of a crushing event. CLE can be calculated using Eq. (4).

(4) $$\text{CLE}=\frac{P_{\text{mean}}}{P_{\max}}\;x\;100$$

#### 2.4.3 Bending test

Three-point quasistatic bending tests are commonly used to determine the response or behavior of a composite material when subjected to bending loads. In this study, three sets of specimens, namely, PLA, GFRP, and hybrid PLA/GFRP tubes, were subjected to quasistatic bending loads. Carson 50 kN UTM was used to perform the test at a crosshead speed of 1 mm/min.

#### 2.4.4 Hardness test

The hardness test was repeated 5 times for each variation of the test specimens using a Shore D hardness tester as per ASTM D 2240 [62] to determine the hardness value of the specimens.

#### 2.4.5 Surface roughness test

Surface roughness testing is used to evaluate the surface quality of the specimens. PLA, GFRP, and hybrid PLA/GFRP were tested for 5 repetitions, and the surface roughness test was carried out perpendicular to the direction of the fiber winding using the Mitutoyo Surftest SJ210 tool.

## 3 Results and discussion

### 3.1 Specimen fabrication

**Fig. 6:**
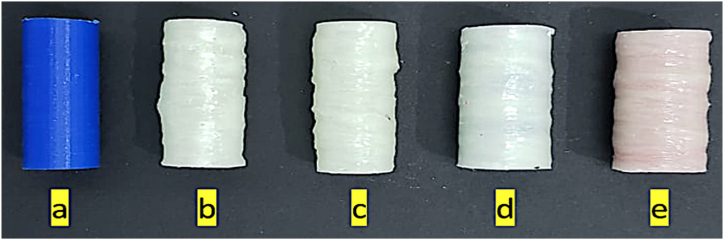
Variation specimens used in this research: **(a)**PLA **(b)** HG **(c)** FG **(d)** HPG **(e)** FPG.

The fabricated specimens are shown in Fig. 6(a–e). The inner diameter of all the specimens was 15.24 mm, and the length was 45.7 mm. The properties of the specimens are given in Table 5.

**Table 5:** The properties of the specimens.

No.	Material	Specimen Code	Methods	Thickness (mm)	Weight (g)	Fiber Volume Fraction (%)
**1**	PLA	PLA	Additive Manufacturing	4.60	5.72	–
**2**	GFRP	HG	Hand Lay-Up	4.73	8.56	0.51
**3**	GFRP	FG	Filament winding	4.89	10.41	0.42
**4**	PLA + GFRP	HPG	Hand Lay-Up	9.32	15.11	0.41
**5**	PLA + GFRP	FPG	Filament winding	9.37	15.49	0.42

### 3.2 Density test

**Fig. 7:**
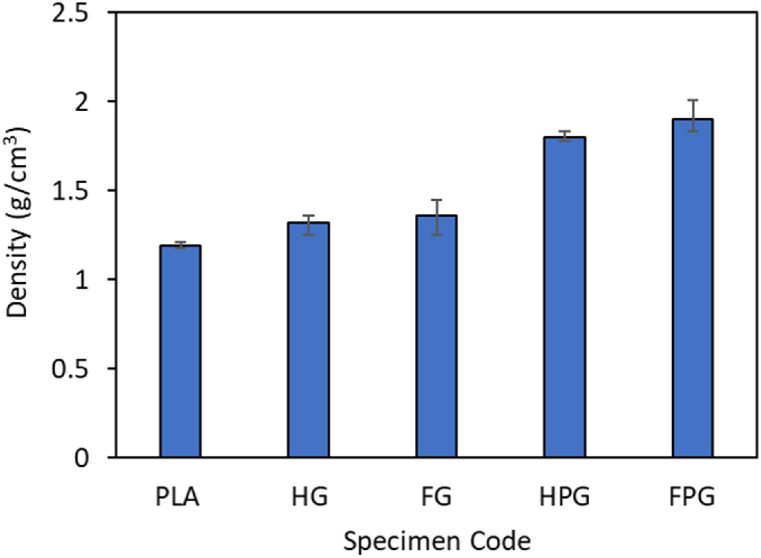
Density test results.

**Fig. 8:**
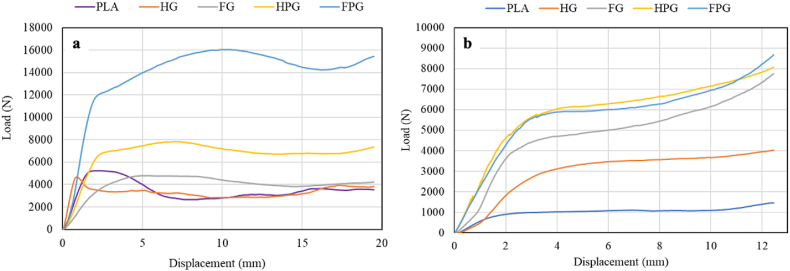
Load‒displacement curves of the **(a)** axial and **(b)** radial compression tests.

The density of each specimen was measured to better understand the relationship between material composition and mechanical performance. These measurements are crucial for analyzing how the integration of different materials, such as PLA and GFRP, influences the structural properties of the composites. Fig. 7 below presents the density data for pure PLA, GFRP, and hybrid PLA/GFRP specimens, which were produced using both the hand lay-up and filament winding methods. The results of these tests show the density data for each specimen, as presented in Fig. 7. The density of the pure PLA material is 1.19 g/cm³. The density values of the GFRP material produced using the hand lay-up and filament winding methods are 1.32 g/cm³ and 1.36 g/cm³, respectively. The hybrid PLA/GFRP specimens have higher density values. Those produced using the hand lay-up method have a density of 1.80 g/cm³, while those made with the filament winding method have a density of 1.9 g/cm³. These results suggest that increasing the GFRP content directly correlates with an increase in density, which occurs glass fibers have a higher density than PLA. The increased density, particularly in the FPG specimens, is also reflected in the mechanical performance during the compression tests. The FPG specimens, with the highest density of 1.9 g/cm³, demonstrate significantly higher peak loads compared to the pure PLA and GFRP specimens. This indicates that improved compaction and material distribution, as shown by the increased density, contribute to a greater load-bearing capacity. This is evident in the load-displacement curves as shown in Fig. 8.

Furthermore, the higher density of the hybrid specimens enhances their energy absorption capabilities. For example, the FPG specimens show superior energy absorption values of 262.18 J in axial loading, as demonstrated in the crashworthiness performance tests as shown in Fig. 11(a). This improvement in energy absorption can be attributed to the higher density achieved through the filament winding process. The increased density allows the material to absorb and dissipate more energy under compressive loads. Additionally, the specific energy absorption (SEA) results follow a similar trend as shown in Fig. 12. The FPG specimens exhibit the highest SEA values, showing that the increase in GFRP content not only enhances the material's density but also improves its efficiency in energy absorption per unit mass. This makes the hybrid composite more effective in crashworthiness applications. Based on these results, it can be concluded that integrating GFRP significantly increases the density and overall mechanical performance of the hybrid PLA/GFRP composite.

### 3.3 Load-displacement response and failure analysis

The load-displacement curve during the compression test is divided into three main parts: crushing, post-crushing, and material densification. The results of the axial and radial loading tests are shown in Fig. 8(a–b). The curve shows that the variety of materials and methods used to manufacture the specimen significantly affect the load exerted by the machine when compressing the specimen. Three material variations were tested using two different manufacturing methods. Specimens manufactured with the filament winding method produce a greater load than those made with the hand lay-up method. The axial and radial compression tests indicate that the FPG (Filament-Winding PLA/GFRP hybrid) samples have the highest load-displacement ratio. This suggests that hybrid PLA/GFRP outperforms the other specimens.

These findings align with previous studies, such as Li et al. [14], which highlight that hybrid composites like PLA/GFRP offer an optimal balance between mechanical strength and environmental benefits. This balance is particularly important in applications that require lightweight materials and high crashworthiness. Fig. 8 shows that specimens made using the filament winding approach perform better than those made with the manual lay-up method. This improvement is likely due to better compaction and resin distribution achieved through the filament winding process. Table 4 confirms this, showing that filament winding specimens have a heavier average weight. Fig. 7 also reveals that they exhibit higher density compared to the hand lay-up specimens. However, in radial compression tests, both methods yield similar results, suggesting that radial stiffness is less affected by the manufacturing process. These results are consistent with Ge et al. [10], who found that hybridizing PLA with glass fibers significantly improves the composite's load-bearing capacity under both axial and radial loading.

In general, circular specimens tested under radial compression typically form four hinge regions. This has been observed in studies by Zha et al. [63], Ma et al. [55], and Baroutaji et al. [11]. These regions become stress-concentration centers, which influence failure modes. The difference between axial and radial loading results is mainly due to the variation in specimen stiffness. Fig. 8(a) shows that PLA-GFRP hybrid specimens produced with filament winding perform better under axial loading. This is likely due to the improved bonding between PLA, glass fibers, and epoxy resin. However, Fig. 8(b) shows that in radial compression tests, both filament winding and hand lay-up methods result in similar stiffness values, suggesting that radial stiffness is less sensitive to the manufacturing process. Compared to pure PLA and GFRP specimens, the hybrid PLA/GFRP composite shows superior performance, particularly in axial compression. This confirms the synergistic effect of combining PLA's biodegradability with GFRP's mechanical strength. These improvements make the hybrid composite a strong candidate for use in industries such as automotive and aerospace, where both strength and sustainability are critical.

To achieve optimal mechanical performance in hybrid PLA/GFRP composites, several key parameters of the FDM-filament winding process were optimized. The layer thickness for the filament-winding hybrid specimens was set at 9.37 mm, ensuring proper fiber embedding within the PLA matrix to enhance structural rigidity. The fiber volume fraction was maintained at 0.42 %, balancing mechanical strength with lightweight characteristics. These optimized conditions led to uniform fiber distribution, minimized defects, and resulted in composites with superior load-bearing capacity, enhanced energy absorption, and higher specific energy absorption (SEA). Consequently, the hybrid PLA/GFRP composites demonstrated improved tensile strength and crashworthiness compared to specimens produced with different methods or parameters, highlighting the effectiveness of the optimized FDM-filament winding process.

### 3.4 Crashworthiness performance

Crashworthiness is the ability of a material or structure to absorb the energy received by the material under a given load. Several crashworthiness parameters can be determined after the specimen is subjected to compression testing: peak load, energy absorption, mean crush load, crushing load efficiency, and specific energy absorption.

#### 3.4.1 Peak load

One of the parameters used to measure the crashworthiness performance of materials is the peak load. It helps in determining the initial process of failure and the safety of the structures. The peak load presented in this study is the maximum value for each variation of the compression test in the axial and radial directions.

**Fig. 9:**
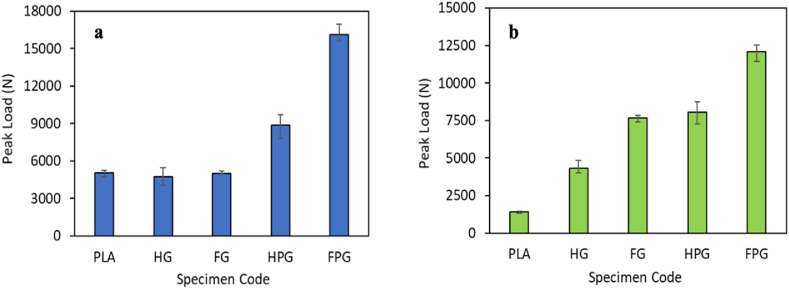
Peak load in the **(a)** axial direction and **(b)** radial direction.

Fig. 9(a–b) shows the variation in the peak load of each material under axial and radial compression tests. Based on the figure, the variation in the materials and methods used for specimen manufacturing affects the peak load of each variation. The peak load process begins with the composite tube becoming elastic, which is accompanied by an increase in the load released by the UTM machine until the maximum loading point and then a significant decrease. The difference in peak load values between specimens is influenced by the geometry and tube material used [73]. The peak load of the specimens fabricated using the filament winding method is greater than that of the specimens fabricated using the hand lay-up method. The GFRP specimens fabricated using the filament winding method had a peak load value of 5013 N, while those fabricated using the hand lay-up method had a peak load value of 4740 N. There was an increase in the peak load value of 5.75 % from the specimens fabricated with the filament winding method compared with the hand lay-up method. Another material variation is the combination of PLA and GFRP. The peak load of the material fabricated using the filament winding method is 16130.5 N, while that of the material fabricated using the hand lay-up method is 8847.33 N. There is an increase in the peak load of 82.32 %. Based on the figure in the radial direction, the GFRP specimens fabricated using the filament winding method have a peak load of 7665.33 N, while those fabricated using the hand lay-up method have a value of 4344.00 N. There is an increase in the peak load of 76.45 %. Another variation of the material from the test is the combination of PLA/GFRP. The peak load of the material fabricated using the filament winding method is 12077.33 N, while that of the material fabricated using the hand lay-up method is 8039.33 N. There is an increase in the peak load of 50.23 %.

#### 3.4.2 Mean crush load

**Fig. 10:**
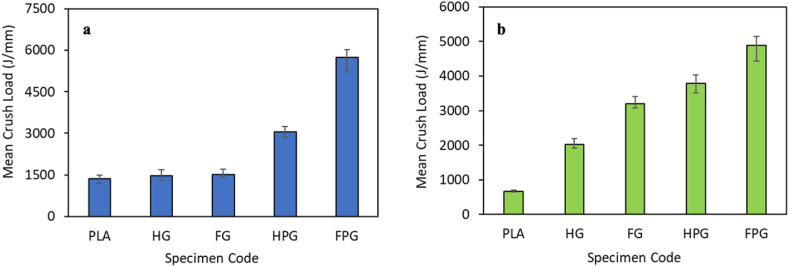
Mean crush load in the **(a)** axial direction and **(b)** radial direction.

The mean crush load is the average value of the load contained in the postcrushing phase, where this value is a good indicator of the overall energy absorption ability of the tested specimen. The average impact load can be calculated by dividing the total absorbed energy by the length of the specimen under test. The results of the mean crush load for each specimen under axial and radial compression tests are shown in Fig. 10(a–b).

**Fig. 11:**
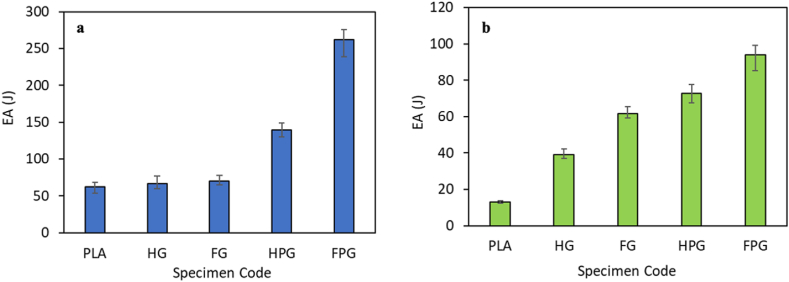
Energy absorption in the **(a)** axial direction and **(b)** radial direction.

**Fig. 12:**
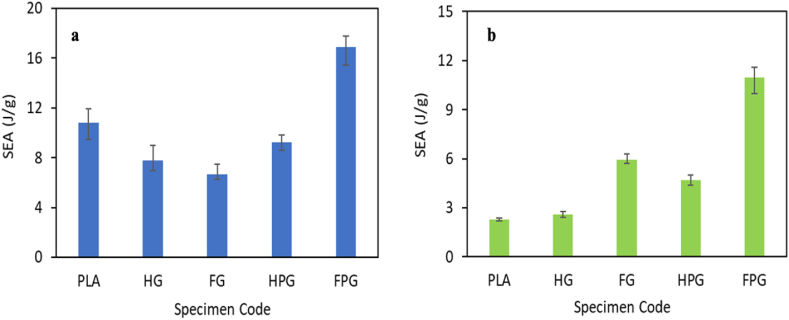
Specific energy absorption in the **(a)** axial direction and **(b)** radial direction.

Based on the figure, the material variation and the method used for specimen manufacturing affect the mean crush load for each variation. The mean crush load of specimens fabricated using the filament winding method is greater than that of specimens fabricated using the hand lay-up method. GFRP specimens fabricated using the filament winding method have a mean crush load of 1521.50 J/mm, while those fabricated using the hand lay-up method have a mean crush load of 1460.53 J/mm. There is an increase in the mean crush load of 4.17 % of the specimens fabricated using the filament winding method compared to the hand lay-up method. Another material variation is the combination of PLA and GFRP. The mean crush load of the material fabricated using the filament winding method was 5734.43 J/mm, while that of the material fabricated using the hand lay-up method was 3050.53 J/mm. There is an increase in the mean crush load of 87.98 %. The increase in the mean crush load of the PLA/GFRP hybrid composite with pure PLA material is 323.47 %. This value shows that the addition of glass fibers to the PLA material can improve the crashworthiness performance of the material, as seen from the mean crush load parameter. GFRP specimens fabricated using the filament winding method have a mean crush load of 3207.19 J/mm, while those fabricated using the hand lay-up method have a value of 2027.00 J/mm. There is an increase in the mean crush load of 58.22 %. Another material variation from this study's existing tests is PLA/GFRP. The mean crush load of the material fabricated using the filament winding method was 4886.75 J/mm, while that of the material fabricated using the hand lay-up method was 3787.70 J/mm. There is an increase in the mean crush load of 29.02 %.

#### 3.4.3 Energy absorption

Energy absorption can be calculated from the area under the load‒displacement curve of the compression test results.

Fig. 11(a–b) shows the variation in the energy absorption of each specimen tested via axial and radial compression tests. Based on the figure, the material variation and the method used for specimen manufacturing affect the EA value of each variation. The EA of the specimens fabricated using the filament winding method was greater than that of the specimens fabricated using the hand lay-up method. The GFRP specimens fabricated using the filament winding method had an EA value of 69.52 J, while those fabricated using the hand lay-up method had an EA value of 66.77 J. There was an increase in the EA value of 4.12 % from the specimens fabricated using the filament winding method with the hand lay-up method. Another material variation is the combination of PLA and GFRP. The EA of the material fabricated using the filament winding method is 262.18 J, while that of the material fabricated using the hand lay-up method is 139.47 J. The resulting increase in the EA is 87.98 %. The increase in the EA of the PLA/GFRP hybrid composite with the pure PLA material is 323.49 %. This value shows that adding glass fibers to the PLA material can improve the crashworthiness performance of the material, as seen from the energy absorption parameter. The EA value of the compression test results in the radial direction for the GFRP specimens fabricated using the filament winding method is 61.71 J, while those fabricated using the hand lay-up method are 39.00 J. There is an increase in the EA value of 58.23 %. Another variation of material from the existing tests in this study is PLA/GFRP. The EA of the material fabricated using the filament winding method is 94.02 J, while that of the material fabricated using the hand lay-up method is 72.88 J. An increase in the EA of 29.01 % is observed.

#### 3.4.4 Specific energy absorption

The specific energy can be calculated by dividing the absorption energy by the mass of each specimen being tested.

Fig. 12(a–b) shows the specific energy absorption for each specimen tested via axial and radial compression tests. Based on the figure, the variation of materials and methods used for specimen manufacturing affects the SEA value of each variation. Fig. 12 (a) shows that GFRP specimens fabricated using the filament winding method have an SEA of 6.68 J/g, while those fabricated using the hand lay-up method have an SEA of 7.80 J/g. Another material variation is the combination of PLA and GFRP materials. The SEA of the material fabricated using the filament winding method is 16.92 J/g, while that of the material fabricated using the hand lay-up method is 9.23 J/g. Fig. 12(b) shows the SEA values of the radial compression test results. The SEA of the GFRP specimens fabricated using the filament winding method was 5.93 J/g, while that of the GFRP specimens fabricated using the hand lay-up method was 2.58 J/g. Another material variation from this study's existing tests is PLA/GFRP. The SEA of the material fabricated using the filament winding method is 10.98 J/g, while that of the material fabricated using the hand lay-up method is 4.70 J/g. According to this research, adding PLA material as a mandrel to glass fiber composites significantly increased their ability to absorb energy compared to that of composites made entirely of glass fibers. Additionally, the presence of PLA made the deformation process of the composites more stable. These results align with the findings of Quanjin et al. [15], who demonstrated that adding PLA material to fiber-based composites increases the specific energy absorption value while stabilizing the damage process. This is made possible by the lightweight properties of PLA and its strong link with the reinforcing fiber.

Comparing the crashworthiness performance of the hybrid PLA/GFRP composites with traditional PLA and GFRP composites further emphasizes the advantages of the hybrid material. The hybrid PLA/GFRP composites exhibited significantly higher specific energy absorption (SEA) values compared to pure PLA, which inherently lacks the mechanical strength required for high crashworthiness applications. In contrast, while GFRP alone offers strong mechanical properties, the addition of PLA enhances the stability of the damage process, as confirmed by Quanjin et al. [15]. The hybrid material also offers a weight reduction, an important factor in automotive and aerospace applications, without compromising crashworthiness performance. This combination of enhanced energy absorption, damage stability, and lightweight properties makes hybrid PLA/GFRP composites a more suitable choice for impact-resistant applications compared to pure PLA and GFRP composites.

#### 3.4.5 Crushing load efficiency

The crushing load efficiency is the ratio of the average impact load to the maximum load issued by the UTM machine when pressing the test specimen.

This efficiency value is used to evaluate the stability of the structure when subjected to collisions [[64], [65], [66]] and the performance of the absorber used [67]. The higher the efficiency of the impact load is, the greater the energy absorption. This can reduce the number of victims involved in a collision [53].

**Fig. 13:**
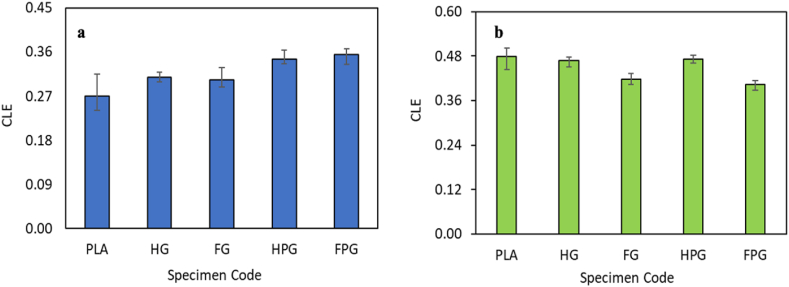
Crushing load efficiency in the **(a)** axial direction and **(b)** radial direction.

Fig. 13(a–b) shows the crushing load efficiency (CLE) for each variation of the specimen tested via axial and radial compression tests. Based on the figure, the material variation and the method used for specimen manufacturing affect the CLE value of each variation. Fig. 13(a) shows that the CLE of specimens fabricated using the hand lay-up method was greater than that of specimens fabricated using the filament winding method. The CLE of GFRP specimens fabricated using the hand lay-up method was 0.31, while that of GFRP specimens fabricated using the filament winding method was 0.30. Another material variation is the combination of PLA and GFRP. The CLE of the material fabricated using the filament winding method is 0.36. Fig. 13(b) shows the CLE values of the radial compression test results. Based on the figure, the GFRP specimens fabricated using the filament winding method have a CLE value of 0.42, while those fabricated using the hand lay-up method have a value of 0.47. Another variation of material from the existing tests in this study is PLA/GFRP. The CLE of the material fabricated using the filament winding method was 0.40, while that of the material fabricated using the hand lay-up method was 0.47.

### 3.5 Bending testing

**Fig. 14:**
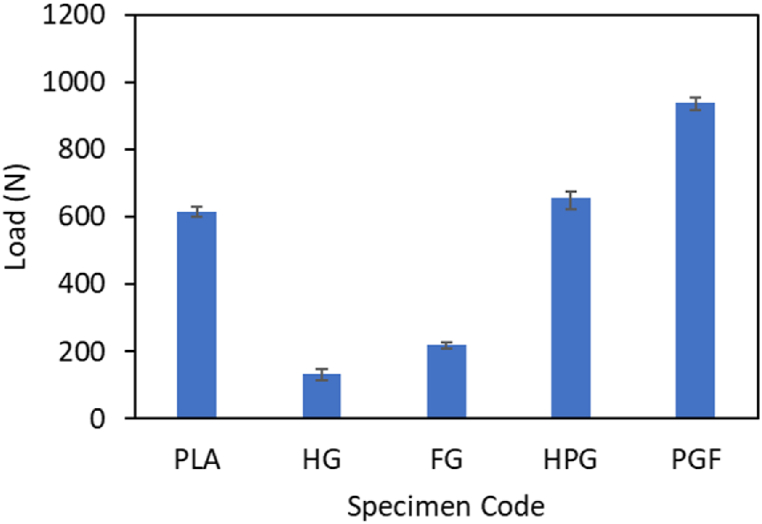
Maximum load on the bending test.

The results obtained are the maximum force data issued by the machine to bend the specimens. The results of these tests are shown in Fig. 14. Based on this figure, the material with the HG specimen code has the lowest buckling force value of 132.33 N, followed by the specimen with the FG code, whose buckling force value is 218 N. The highest buckling force value is found in the specimen with the code PGF, whose value is 939.33 N.

### 3.6 Hardness

**Fig. 15:**
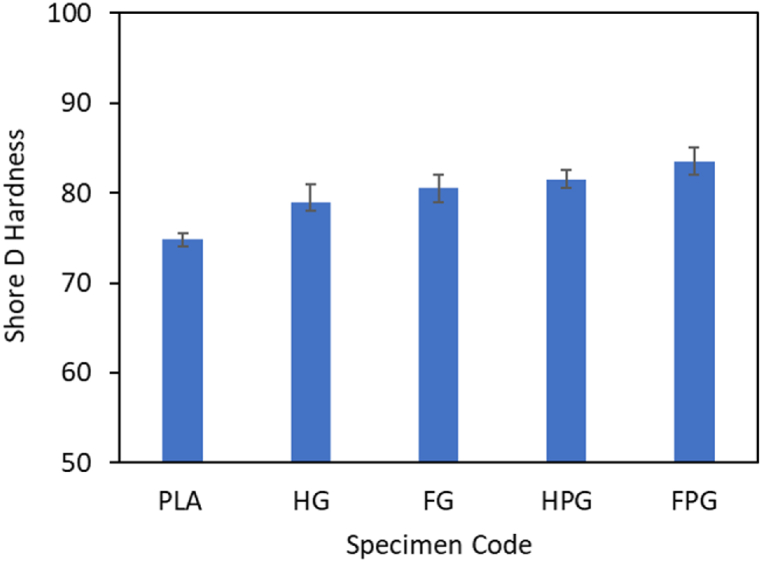
Shore D hardness.

Hardness testing is used to determine the hardness of the specimens. Based on the graph shown in Fig. 15, the hardness of the PLA tube has the lowest value among the other material variations, which is 74.9.

For tubes made of GFRP only, the hardness values for tubes made by hand lay-up and filament winding methods are 79 and 80.5, respectively. Another variation found in this study is the combination of PLA and GFRP. The hardness values of the tubes made using a combination of the two materials made by hand lay-up and filament winding methods are 81.5 and 83.5, respectively. This value is the highest among the other material variations. Based on these results, adding GFRP material to PLA can increase the hardness of the material by 8.85 % for tubes fabricated by the hand lay-up method and 11.48 % for tubes fabricated by the filament winding method.

### 3.7 Surface roughness

**Fig. 16:**
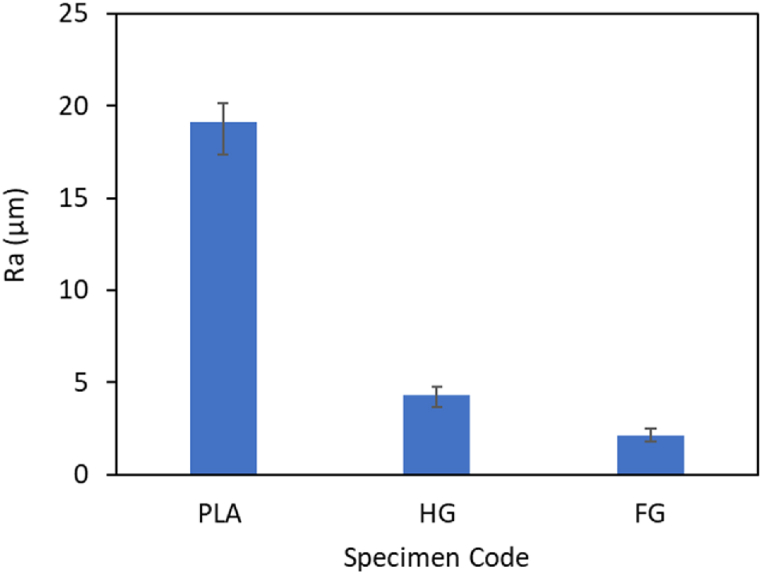
Surface roughness.

Surface roughness testing was carried out to determine the surface quality of the specimens. The test was carried out using a surface roughness tool. There were two variations of tube specimens that were tested, namely, tubes with pure PLA and those with GFRP materials. The surface roughness test in this study was carried out perpendicular to the direction of the fiber winding. A summary of the surface roughness test results from the two variations is shown in Fig. 16. GFRP materials have two different surface roughness values because they are fabricated using two different methods. Based on the figure, the PLA material has the highest Ra value or surface roughness compared to the other materials. This is because the testing process is carried out perpendicular to the direction of the PLA tube mold, so the roughness is high. Concerning the variation in GFRP material, the surface roughness of the tube fabricated by the filament winding method was lower than that of the tube fabricated by the hand lay-up method. This is because, in the hand lay-up method, the lamination process is carried out using a brush so that the surface of the glass fiber wetted with resin becomes uneven.

### 3.8 Failure form

The failure modes of the PLA, GFRP, and PLA/GFRP hybrid composite tubes were investigated in this study. Several forms of failure modes can occur in specimens under axial and radial compression loading: matrix cracking, fiber breakage, folding, etc.

**Fig. 17:**
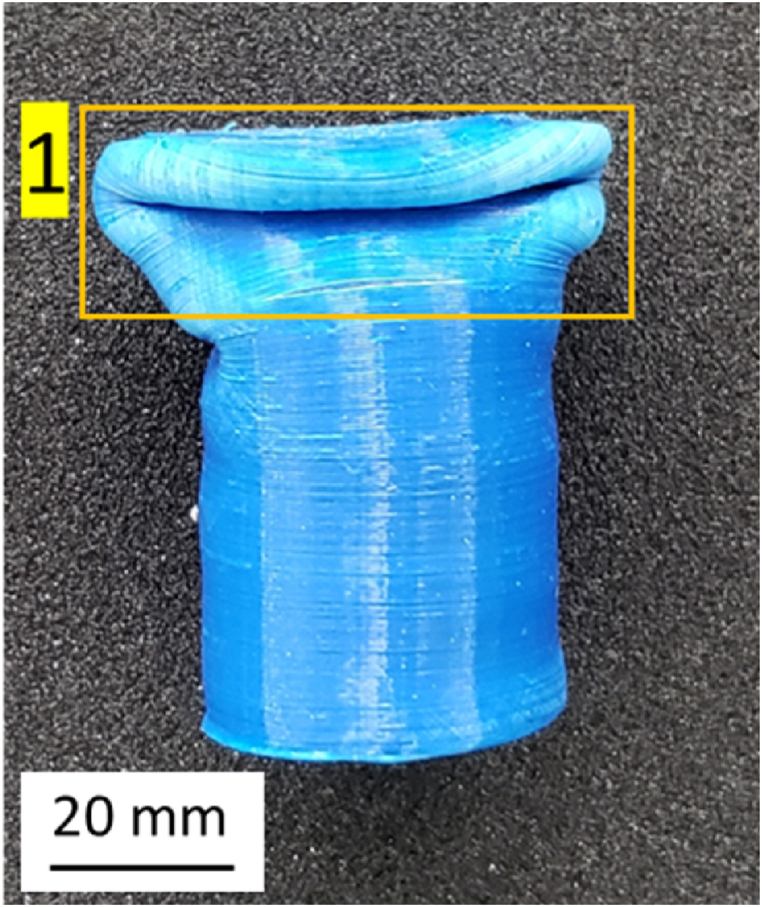
Folding on PLA material.

The form of failure that appears in the PLA material is folding. This folding occurs because the PLA is plastically deformed under a compressive load. The deformation causes the accumulation of material, as shown in Fig. 17.

**Fig. 18:**
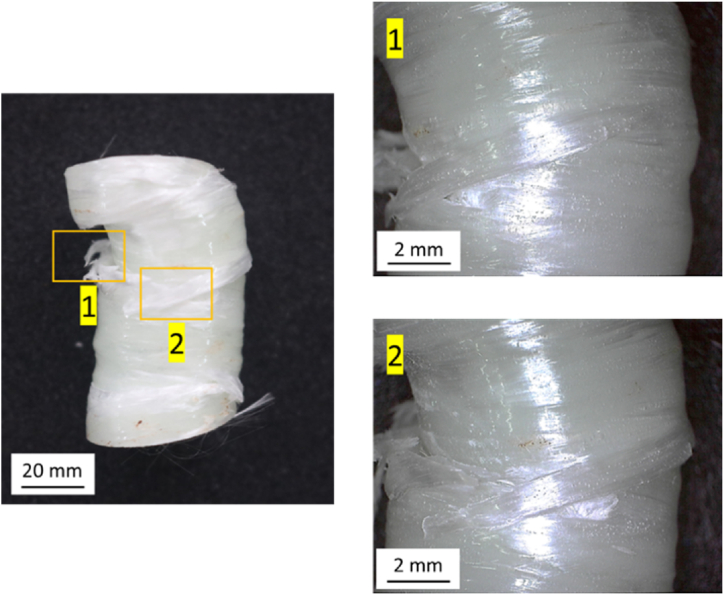
The failure mode of GFRP materials fabricated by the hand lay-up method.

Fig. 18 shows the failure mode of the GFRP material fabricated by the hand lay-up method. The failure mode in this variation is matrix cracking, shown in number 1, and fiber breakage, as shown in number 2. Matrix cracking is a form of failure that first occurs in a composite structure. Cracking that appears on the specimen reduces the mechanical strength of the material. Matrix cracking can occur due to the inability of the matrix to withstand the strain caused by compressive loading. The second form of failure mode is fiber breakage. Fiber breakage is caused by the inability of the fiber to withstand the load that occurs when the specimen is deformed when subjected to compressive loading.

**Fig. 19:**
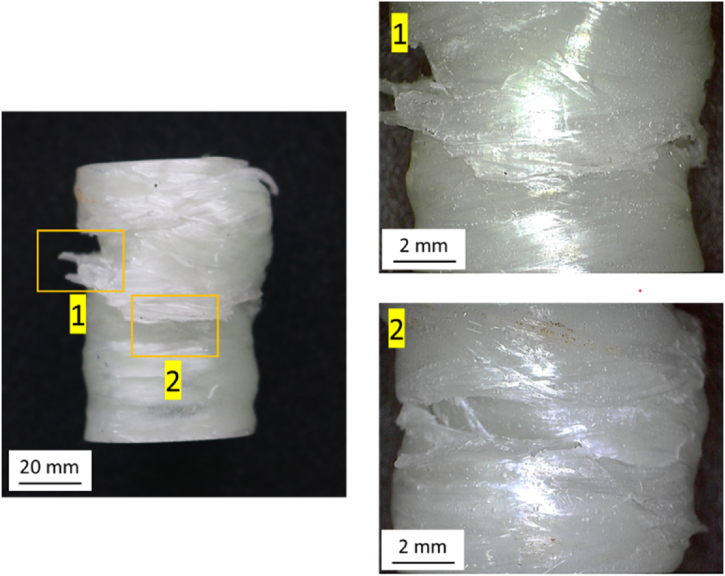
The failure mode of GFRP materials fabricated by the filament winding method.

Fig. 19 shows the failure mode of the GFRP material fabricated by the filament winding method. Two failure modes appear in this variation: matrix cracking, which is indicated by number 1, and fiber breakage, which is indicated by number 2.

**Fig. 20:**
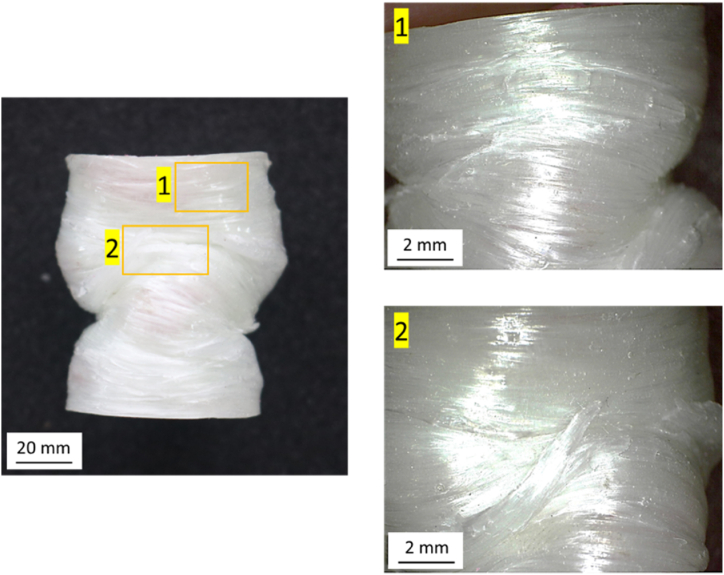
The failure mode of the PLA/GFRP material fabricated by hand lay-up.

Fig. 20 shows the failure mode of the PLA/GFRP material fabricated by the hand lay-up method. The primary failure mode observed is matrix cracking caused by compressive loading. Matrix cracking occurs when the matrix cannot withstand the strain from the compressive force. This type of cracking often initiates failure in hybrid composites, especially in hand lay-up specimens, where the bond between the PLA matrix and GFRP layers may be weaker than in filament-wound specimens. Once matrix cracking begins, it can spread through the composite, leading to more severe failure, such as delamination between the PLA and GFRP layers. This delamination weakens the composite's overall structure, reducing its ability to absorb further impact energy. These findings align with Sethi et al. [68], who noted that matrix cracking often leads to delamination, particularly in hybrid composites where the matrix material is more brittle than the reinforcing fibers.

**Fig. 21:**
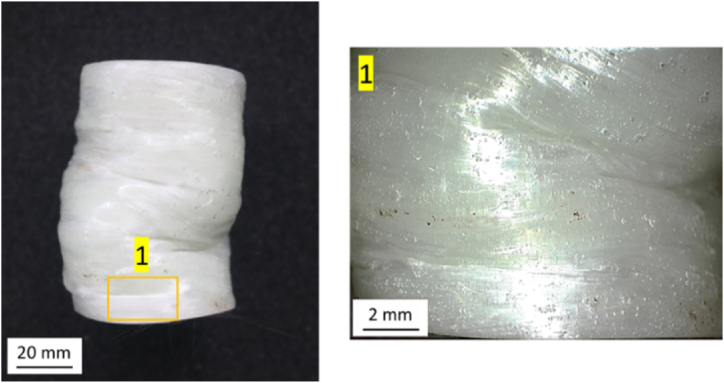
The failure mode of the PLA/GFRP material fabricated by the filament winding method.

Fig. 21 shows the failure mode of the PLA/GFRP material fabricated by the filament winding method. The observed failure modes include matrix cracking, as indicated by number 1, and fiber breakage, as indicated by number 2. Matrix cracking typically occurs first in a composite structure and reduces the material's properties. This cracking happens when the matrix cannot handle the strain from compressive loading. However, in filament-winding specimens, the stronger bond between layers lowers the risk of delamination compared to hand lay-up specimens. The second failure mode is fiber breakage, which occurs when the fibers cannot withstand the load as the specimen deforms under compressive stress. This is consistent with the research by Liu et al. [69], who found that fiber breakage often follows matrix cracking, especially when the matrix has already failed to support the fibers. Quanjin et al. [15] also noted that adding PLA to fiber-reinforced composites helps stabilize the damage process, reducing the severity of fiber breakage by improving energy dissipation.

**Fig. 22:**
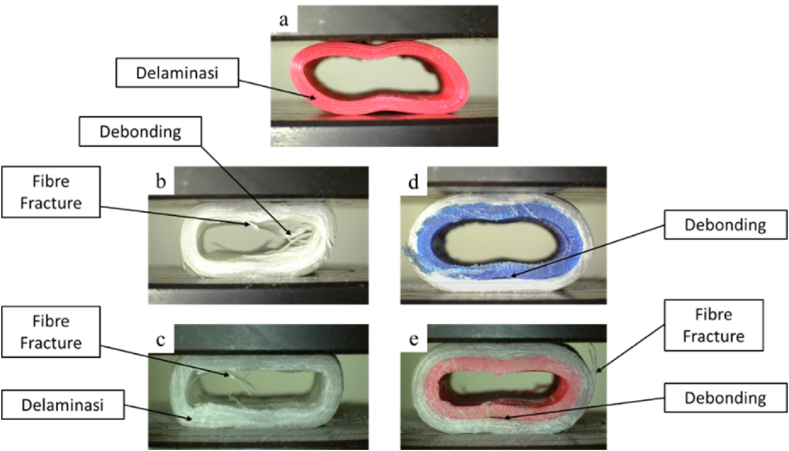
The failure mode of the radial compression test: **(a)** PLA, **(b)** HG, **(c)** FG, **(d)** HPG, and **(e)** FPG.

Fig. 22(a–e) shows the form of failure that appears when the material is subjected to radial compressive loads. The form of failure that appears in the PLA material is delamination, caused by the weak bonding between the layers in the material. In GFRP materials, the failure modes that occur are fiber fracture, debonding, and delamination, whereas in hybrid materials, the failure modes that occur are fiber fracture and debonding. The failure process of the PLA, GFRP, and PLA/GFRP hybrid materials under radial compression testing begins with the specimen entering the elastic region of the material. The delamination process in PLA materials is caused by the weak bonding between layers when subjected to a compressive load. In GFRP materials, as the displacement of the pressure plate increases, several forms of failure, such as debonding, fiber fracture, and debonding, appear. Fiber fractures in these materials occur near the plastic hinge area, as in the study by Zha et al. [63]. The form of failure in the hybrid material is the same as that in the pure GFRP material. The combination of PLA and GFRP materials produces a better failure pattern compared to GFRP materials, where it can be seen that there is debonding between the glass fiber material and the matrix used. In the hybrid material, the debonding process of the material does not occur because there is a PLA material that prevents failure.

The results of the axial and radial compression tests found in this study are similar to those of the studies conducted by Supian et al. [51] and Hu et al. [52]. The trend of increasing peak loads of PLA, GFRP, and hybrid PLA/GFRP is similar to that of the peak loads from research conducted by Supian et al. The failure process of the axial compression-tested hybrid specimens is greater than that of the pure GFRP and PLA materials. This study provides insight into the properties of PLA/GFRP hybrid composite materials, which can have potential future applications as materials with excellent crashworthiness properties.

The use of hybrid PLA/GFRP composites presents significant economic and sustainability advantages. PLA, being a biodegradable and cost-effective polymer, reduces material costs and supports environmentally friendly production. This makes it an attractive option in applications where both performance and sustainability are priorities. Additionally, the manufacturing process, particularly when using additive manufacturing techniques like FDM-filament winding, can minimize material waste and further reduce costs. However, limitations such as PLA's lower thermal resistance and mechanical strength compared to more expensive polymers should be considered when applying this material in high-performance settings. Overall, hybrid PLA/GFRP composites offer a balance of cost-efficiency, mechanical performance, and environmental benefits, making them a viable alternative in various industrial applications.

## 4 Conclusion

The study demonstrated that hybrid PLA/GFRP composites fabricated using the FDM-filament winding technique exhibit superior mechanical properties and enhanced crashworthiness performance, making them a promising material for applications demanding lightweight and energy-absorbing structures. Based on the results, the following conclusions can be drawn:

• The addition of fiberglass material to PLA can increase the crashworthiness performance of the material. The parameters investigated in this study were the peak load, energy absorption, mean crush load, crushing load efficiency, and specific energy absorption. The values were obtained from the results of compression tests in the axial and radial directions. The highest peak loads from the compression test results in the axial and radial directions are found in the variation with the FPG specimen codes, whose values are 16130.50 N and 12077.33 N, respectively. The highest mean crush load is found in the material variation with the FPG specimen code, whose value is 5734.43 J/mm for axial loading and 4886.75 for radial loading. The highest EA value is found in the material variation with the FPG specimen code, whose value is 262.18 J for axial direction loading and 94.02 for radial direction loading. The highest SEA value is found in the material variation with the specimen code FPG, whose value is 16.92 J/g for axial loading and 10.98 for radial loading. The last parameter is CLE, which has the highest value for material variation with the FPG specimen code for axial direction loading, with a value of 0.56, and the PLA specimen code for radial loading, with a value of 0.38.
• The maximum force required by the UTM machine to bend the specimen is the highest in the material variation with the specimen code FPG, which has a value of 939.33 N, while the lowest bending force is found in the material variation with the specimen code FG, which has a value of 218 N.
• The hardness of the PLA tube has the lowest value among the other material variations, which is 74.9. For tubes made of GFRP only, the hardness values for tubes made by hand lay-up and filament winding methods are 79 and 80.5, respectively. The hardness values of the tubes made using a combination of PLA/GFRP by hand lay-up and filament winding methods are 81.5 and 83.5, respectively.
• The surface roughness test, which was carried out by comparing 3 variations of the specimen, showed that the surface roughness of the PLA material was greater than that of the other materials.
• The density of the pure PLA material was measured to be 1.19 g/cm^3^. The density values of the GFRP material produced using the hand lay-up and filament winding methods are 1.32 g/cm^3^ and 1.36 g/cm^3,^ respectively. The last variation of the specimen is a hybrid between PLA and GFRP, whose density values of the hybrid material are 1.80 g/cm^3^ and 1.9 g/cm^3,^ respectively, printed using hand lay-up and filament winding.

The results from the study show that the hybrid PLA/GFRP outperforms other materials in terms of energy absorption. This suggests that the hybrid PLA/GFRP fabricated using FDM-filament winding has great potential as a crash-worthy material for future applications.

## CRediT authorship contribution statement

**Ariyana Dwiputra Nugraha:** Writing – review & editing, Writing – original draft, Visualization, Investigation, Formal analysis, Data curation. **Rahmad Kuncoro Adi:** Writing – review & editing, Writing – original draft, Validation, Investigation, Formal analysis, Data curation. **Vishnu Vijay Kumar:** Writing – review & editing, Writing – original draft, Visualization, Validation, Methodology, Investigation, Data curation. **Arif Kusumawanto:** Writing – review & editing, Writing – original draft. **Budi Prawara:** Writing – review & editing, Writing – original draft. **Endro Junianto:** Writing – review & editing, Writing – original draft. **Muhammad Fathul Hikmawan:** Writing – review & editing, Writing – original draft. **Muhammad Akhsin Muflikhun:** Writing – review & editing, Writing – original draft, Supervision, Resources, Project administration, Methodology, Conceptualization.

## Data availability statement

The datasets generated during and/or analyzed during the current study are available from the corresponding author upon reasonable request.

## Declaration of competing interest

The authors declare that they have no known competing financial interests or personal relationships that could have appeared to influence the work reported in this paper.
